# Anesthetic management in a patient with Arnold‐Chiari malformation type 1,5: A case report

**DOI:** 10.1002/ccr3.5194

**Published:** 2022-02-04

**Authors:** Antonio Coviello, Ludovica Golino, Concetta Posillipo, Annachiara Marra, Andrea Tognù, Giuseppe Servillo

**Affiliations:** ^1^ Department of Anesthesiology and Intensive Care Medicine Policlinico – Federico II University Hospital Napoli Italy; ^2^ Department of Anaesthesia and Postoperative Intensive Care Istituto Ortopedico Rizzoli Bologna Italy

**Keywords:** ACM‐1.5, arnold‐chiari malformation, case report, epidural anesthesia, neuraxial

## Abstract

A 42‐year‐old male patient with Arnold‐Chiari malformation type 1,5 (ACM‐1,5) came to implant a hip prosthesis. He underwent a previous general anesthesia, with difficult airway management and complication in awakening. In this second surgery, an extradural approach was preferred to keep intracranial pressure and hemodynamics stable.

## Graphical abstract

Scientific literature is not very rich in information regarding the best anesthesiological approach to patients with Arnold‐Chiari malformation type II (ACM‐II). We therefore wanted to provide our experience with an ACM‐II patient.

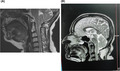

## 1 INTRODUCTION

Arnold‐Chiari malformation type 1,5 (ACM‐1,5) is a developmental abnormality of the brainstem consisting in protrusion of cerebellar tonsils, medulla oblongata, and 4th ventricle into cervical spinal canal, usually associated with myelomeningocele.^1^ McClone and Knepper speculated this open neural tube as a cause of ACM‐1,5 malformations, because it leads to leakage of cerebrospinal fluid resulting in a fourth ventricle that is unable to maintain distension. The continued collapse of the fourth ventricle in utero results in a hypoplastic posterior fossa and cerebellar tonsillar herniation.^2^ Clinical symptoms are headaches, neck pain, apnea, inspiratory wheeze or stridor, breath holding, retrocollis or opisthotonous, irritability, aspiration pneumonia, dysphagia, dysarthria, nystagmus, strabismus, and quadriparesis with hypotonia. Sleep apnea, prolonged expiratory apnea with cyanosis, and aspiration pneumonia are other potential breathing disorders associated with ACM‐1,5.^3^ The main diagnostic test is magnetic resonance imaging (MRI) to evaluate neuroanatomy. If fetal ventriculomegaly is present, the diagnosis of ACM‐1,5 can be made with fetal ultrasound or fetal MRI in some cases.^4^ Patients who have few symptoms can be managed medically with muscle relaxants, non‐steroidal anti‐inflammatory drugs (NSAIDs), and temporary use of a cervical collar. However, the main treatment for ACM‐1,5 is surgical with the goal of re‐establishing the cerebrospinal fluid (CSF) flow across the craniovertebral junction and relieving pressure on the cerebellum by decompressing the posterior fossa. ACM‐1,5 has a 3% neonatal in‐hospital mortality and 15% 3‐year mortality rate. Those that survive can have increasing motor dysfunction over time. Continued follow‐up for shunt placement evaluation or shunt failure is recommended. Prognosis in the more severe ACM variants is unfavorable, with early death.^5^ Respiratory complications have important prognostic implications. The anesthesiologic management of these patients is certainly not an easy challenge. It is necessary to keep the intracranial pressure as stable as possible, avoid neck movements as much as possible, and keep stable hemodynamic.^1^ There is paucity of literature regarding anesthetic management of patients with ACM‐1,5. The anesthetic concerns with general anesthesia (GA) are related to the risk of autonomic dysfunction, difficult airway management, damage to the spinal cord, and sensitivity to neuromuscular blocking agents. There is risk of increasing intracranial pressure and brainstem compression or herniation leading to hemodynamic and respiratory compromise, due to presence of myelomeningocele, syringohydromyelia, and tethered spinal cord.^6^ On the other side, if intracerebral pressure above the level of the foramen magnum is sufficiently high, the negative spinal pressure that potentially could result during the performance of spinal anesthesia (SA) could cause further cerebellar tonsillar herniation and CSF outflow obstruction at the level of the foramen magnum, resulting in subsequent neurologic deterioration.^7^ We believe to provide an interesting point of view since the anesthesiological scientific literature on this type of patient is still quite sparse today.

## 2 CASE PRESENTATION

**FIGURE 1:**
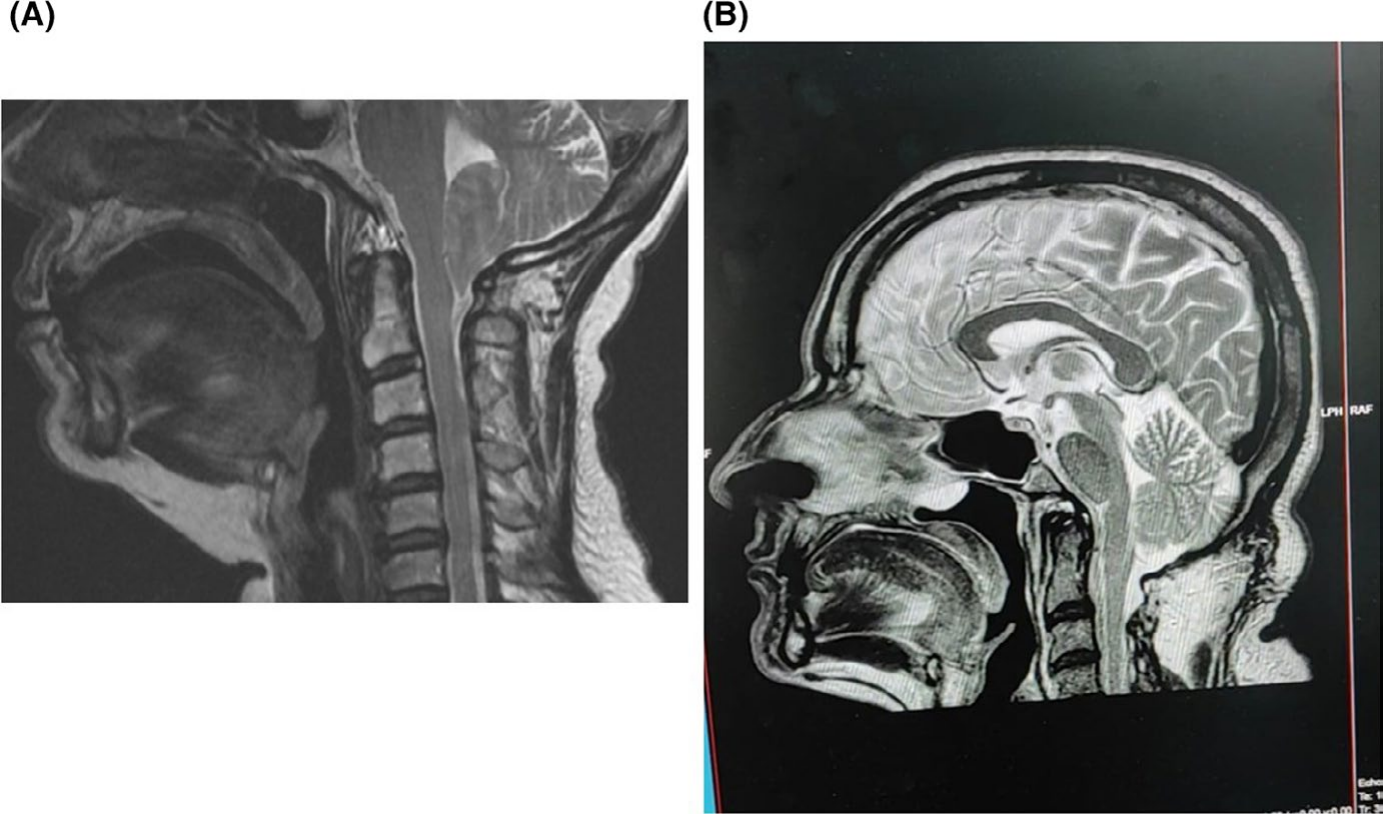
Sagittal magnetic resonance of the patient before (panel A) and after (panel B) posterior fossa decompression surgery for Arnold‐Chiari type 1,5 malformation

The patient was a 42‐year‐old man affected by Arnold‐Chiari malformation classified as type 1,5 (ACM‐1,5) during his first months of life. No other comorbidities. The main pathological finding was cerebellar tonsillar herniation (as seen in Chiari I malformation) along with caudal herniation of some portion of the brainstem (obex of the medulla oblongata) through the foramen magnum, not associated with myelomeningocele as in most severe cases of ACM‐1,5. Physical examination after birth detected Apgar score of 7, cephalic perimeter of 46 cm, torticollis, scoliosis (a bending of the spine to the left), and a tetra‐pyramidal syndrome. He manifested sleep apnea, dysphagia, nystagmus, and intractable headaches. He was operated in childhood, at the age of 10, with decompression of the posterior cranial fossa (Figure 1). After surgery, he had no more respiratory symptoms, dysphagia, and nystagmus. Our patient was in follow‐up from the rare pathologies center; his clinical condition was stable over time. He occasionally suffered from headaches that he treated with NSAIDs. He had slight alterations in walking, coordination, and balance. The patient came to our orthopedic center to place a right hip spacer 5 months earlier and subsequently came to replace the hip spacer with a definitive prosthesis, because he has had an infection from the previous hip replacement. The hip spacer was implanted under GA, and the patient experimented many apneic/desaturation events during awakening. Awakening was also complicated by nausea and vomiting, hypertensive episodes, and general malaise. Post‐anesthesiology care unit (PACU) was required for the next 48 h. In this second surgery, in multidisciplinary agreement with team of anesthesiologist, neurosurgeons, and orthopedics, and in agreement with the patient himself, it was decided to change the anesthesiological technique and we choose epidural anesthesia. An extradural approach was preferred to avoid the risks associated with both GA and dural puncture in a SA.

In the preoperative valuation, all tests were normal, Apfel score 2 (PONV risk 39%), Altemeier's classification 2, Erasmus Score Risk >3, ASA classification III. In the operating room, the vital parameters were monitored: oxygen saturation in peripheral arterial blood (SpO2), electrocardiogram (ECG), and non‐invasive blood pressure (NIBP). The saturation of the patient at rest oscillated around values of 96%–97%. NIBP was 130/80 mmHg. The rhythm was sinus with a frequency of 75 Bpm. Premedication with midazolam 0.03 mg/kg i.v. and dexametazone 4 mg I.V. was administered. In asepsis, epidural anesthesia was performed with the patient in the sitting position after determining the L3‐L4 interspace with anatomical method, identifying L4 with the bisiliac line. The level of puncture was confirmed by ultrasound counting the vertebrae from the sacrum, in a caudo‐cranial sense. With Tuohy needle (17 Gauge), the epidural space was identified at 5 cm from the skin through the loss of resistance in the water spindle (pre‐filled syringe of sterile saline solution). The peridural catheter was inserted 5 cm in the epidural space (10 cm from the skin). Negative pressure occurred in the catheter and negative aspiration for blood and CSF. Epidural dose test was performed according to recommendations, injecting Lidocaine 2% 3 ml and epinephrine 15 mg through the epidural catheter and waiting 5 min to check for motor blockage or cardiovascular symptoms. The test was negative. The procedure was performed by an expert anesthesiologist. Epidural injection of ropivacaine 0.75% 15 ml was practiced two times, a first 7.5 ml bolus and a second 7.5 ml bolus after 10 min. After 15 min, the anesthesiologic plane was tested through pin‐prick test and ice test, assessing the extension of the sensory block between T10‐S4 with Hollmen grade 4 (loss of sensation). Motor block was rated as Bromage II (the patient was able to move his feet). After 20 min, the surgery began without discomfort for the patient. The surgery lasted for 3 h and 15 min. Two hours after starting the surgery, ropivacaine 0.75% 10 ml was administered. There were no particular surgical complications. During the surgery, the patient remained calm and had no pain, discomfort, or other side effects such as nausea or vomiting. The patient's hemodynamics remained stable. We discharged with a patient intermittent epidural boluses (PIEB) / patient‐controlled epidural analgesia (PCEA) epidural pump with programmed boluses of ropivacaine 0,1% (8 ml per hour) and the possibility of boluses at the patient's request (5 ml per hour, with safety lock for the next 60 min). The patient's vital signs were monitored continuously for the next 48 hours. We carried out a serial check of the patient every 8 h, monitoring pain and the appearance of any side effects such as nausea or vomiting, pruritus, and shivering. On the first day after surgery, the patient used the PCEA twice, administering the additional ropivacaine bolus. He did not use the PCEA on the second day. Pain control was optimal, with visual analogue scale (VAS) 2 in the first day after surgery, VAS = 0 from the second day. There were no side effects. After 12 h, the patient was mobilized by the ward staff, and after 18 h, he was able to stand up and walk. On the fourth day, after neurosurgical consultation, the patient was discharged.

## 3 DISCUSSION

We performed an epidural anesthesia in a patient with ACM‐1,5 to implant a hip prosthesis. Our main goal was to keep intracranial pressure and hemodynamics as stable as possible. We also wanted to avoid the respiratory problems that the patient already manifested in the previous recent GA. In fact, GA can lead to respiratory complications in patients with ACM malformation, considering that they can develop obstructive apnea and central apnea. Obstructive apnea is usually reversed with an optimally functioning ventriculoperitoneal shunt, whereas central apnea does not respond to cervical decompression.^5^ Regards anesthesiological approach, Ramsis et al.^8^ to avoid progression of the herniation due to increased intracranial pressure (ICP) during head and neck movements and laryngoscopy, led to an awake fiberoptic intubation was performed under generous topical anesthesia and defined also that in ACM patients it is important to prevent coughing and bucking during endotracheal extubation at the end of GA. Extubation under deep anesthesia may decrease the risk of coughing, but it may increase the risk of gastric aspiration and sudden apnea episodes.^8^ However, GA can cause systemic hypotension and consequently increased ICP, not desiderable in patients with ACM‐1,5. Starting from these considerations, as the type of intervention allowed us, we preferred neuraxial anesthesia. In particular, we preferred epidural anesthesia because it consented greater hemodynamic stability and because it reduced impact on CSF pressure, since any deliquoration was avoided. With an extradural approach we avoided fluctuations of ICP, we avoided breathing problems and neck movements, the patient was awake and cooperative throughout the intervention and we also avoided opioids administration, reducing the risk of respiratory depression. The alternative choice would be to perform a SA with a 27 gauge Whitacre spinal needle. However, we could not be sure to perform a single, atraumatic puncture, and therefore, we could not predict and quantify the real reflux of liquor. Furthermore, the hemodynamic impact of the SA would have been greater and less controllable compared to epidural anesthesia. Finally, to make sure we had a good anesthetic plan for the duration of the surgery, we would use an adjuvant in subarachnoid space, with further impact on hemodynamics. The procedure for positioning the epidural catheter was carried out by an expert anesthesiologist. The risk of accidental dural puncture was 0.49% like reported in literature. The incidence was related to puncture level of spine and age.^9^ In case of accidental puncture of the dura mater, the catheter would have been positioned in the subarachnoid space and continuous SA would have been conducted. In fact, the insertion of the catheter in the subarachnoid space and the continuous SA reduce the consequences related to the accidental puncture of the dura.^10^ Miqi et al.^11^ affirm that epidural anesthesia might raise the intracranial hypertension when a bolus of local anesthetic is given due to dural compression in the epidural space with a shift of CSF into the cranium. To reduce this risk, our approach involved small doses and slow injection of anesthetic into the peridural space. We decided to use a higher anesthetic concentration (ropivacaine 0.75%), in order to reduce the total injected volume (15 ml). We also injected the anesthetic solution in two times (7.5 ml slow boluses 10 min apart). In support of our anesthesiological management, Margarido et al.^12^ affirm that in patients with post‐traumatic syringomyelia, epidural anesthesia may offer several advantages over SA and GA. These benefits include safety of the airway, reduced rate of incidence of hypotension and deterioration of autonomic neuropathy, and minimal change in the existing CSF pressure relationship if the medication is titrated gradually. We inserted the epidural catheter into the epidural space for 5 cm, as recommended by the literature evidence.^13^ We performed epidural test dose with lidocaine 2% 3 ml and epinephrine 15 mg to rule out accidental placement in the subarachnoid space or in a blood vessel, as recommended by the literature studies.^14^ Lastly, we could practice lumbar plexus block in order to avoid any risk of dural puncture. Bin Mei et al.^15^ used this technique during hip surgery when it was preferable to avoid neuroaxial approach. In our case, we risked to not obtain a long‐lasting anesthesiological coverage; also, we would not obtain adequate coverage of postoperative pain without using opioids, which would increase the risk of respiratory depression. Although a Erector spinae block combined with lumbar plexus block, paravertebral block, and sacral plexus blocks with the support of sedoanalgesia could be performed in this case to minimize the risk of hemodynamic fluctuations and dural puncture related to NA, however the risk of local anesthetic toxicity would be effectively increased considering the total volume and dose administration.^16^, ^17^, ^18^ Epidural anesthesia was therefore our choice because it may offer several advantages over SA and GA in patients with ACM‐1,5. These advantages include avoidance the risk of difficult intubation and unsuccessful airway protection, reduced incidence of hypotension and deterioration of autonomic neuropathy, and minimal change in intracranial pressure if the anesthetic is titrated gradually. Also with the extradural approach, it does not alter the CSF pressure because the dura mater is not punctured and there is no CSF leakage.

## 4 CONCLUSION

Our ACM‐1,5 patient presented good hemodynamic during and after surgery, good respiratory compensation, and optimal pain control in the postoperative period thanks to epidural analgesia. He was able to stand up and walk after 18 h. The successful management of this case suggests that epidural anesthesia can be considered as a first choice for these patients reasoning on the risks‐benefits.

## CONFLICT OF INTEREST

The authors declare that they have no competing interests.

## AUTHOR CONTRIBUTIONS

AC and GS had made substantial contributions to the conception LG and CP had made design of the work. CP had made the acquisition and analysis. LG had made interpretation of data. AC had made the creation of new software used in the work. AM has drafted the work or substantively revised it. All authors read and approved the final manuscript.

## ETHICAL APPROVAL

Not applicable.

## CONSENT

Written informed consent was obtained from the patient for the publication of this case report and any accompanying images. A copy of the written consent is available for review by the Editor‐in‐Chief of this journal.

## Data Availability

Data sharing is not applicable to this article as no datasets were generated or analyzed during the current study. If you do not wish to publicly share your data, please write: “Please contact author for data requests
